# Hormonal modulation of connective tissue homeostasis and sex differences in risk for osteoarthritis of the knee

**DOI:** 10.1186/2042-6410-4-3

**Published:** 2013-02-04

**Authors:** Barbara D Boyan, David A Hart, Roger M Enoka, Daniel P Nicolella, Eileen Resnick, Karen J Berkley, Kathleen A Sluka, C Kent Kwoh, Laura L Tosi, Mary I O’Connor, Richard D Coutts, Wendy M Kohrt

**Affiliations:** 1 Isis Research Network on Musculoskeletal Health, Society for Women’s Health Research, 1025 Connecticut Avenue, NW Suite 601, Washington, DC, 20036, USA; 2 Wallace H. Coulter Department of Biomedical Engineering, Georgia Institute of Technology, 315 Ferst Drive NW, Atlanta, GA 30332-0363, USA; 3 Mayo Clinic, 4500 San Pablo Road, Jacksonville, Florida, USA; 4 University of Pittsburgh and Pittsburgh VA Healthcare System, Pittsburgh, PA, USA; 5 Epidemiology, and Clinical and Translational Science Division of Rheumatology and Clinical Immunology, University of Pittsburgh School of Medicine, University of Pittsburgh Arthritis Institute, Pittsburgh, USA

**Keywords:** Bone, Estrogen, Ligaments, Osteoarthritis, Sex differences, Sex steroids, Tendon, Testosterone

## Abstract

Young female athletes experience a higher incidence of ligament injuries than their male counterparts, females experience a higher incidence of joint hypermobility syndrome (a risk factor for osteoarthritis development), and post-menopausal females experience a higher prevalence of osteoarthritis than age-matched males. These observations indicate that fluctuating sex hormone levels in young females and loss of ovarian sex hormone production due to menopause likely contribute to observed sex differences in knee joint function and risk for loss of function. In studies of osteoarthritis, however, there is a general lack of appreciation for the heterogeneity of hormonal control in both women and men. Progress in this field is limited by the relatively few preclinical osteoarthritis models, and that most of the work with established models uses only male animals. To elucidate sex differences in osteoarthritis, it is important to examine sex hormone mechanisms in cells from knee tissues and the sexual dimorphism in the role of inflammation at the cell, tissue, and organ levels. There is a need to determine if the risk for loss of knee function and integrity in females is restricted to only the knee or if sex-specific changes in other tissues play a role. This paper discusses these gaps in knowledge and suggests remedies.

## Review

### Introduction

The knee comprises a number of tissues (bones, muscle, cartilage, menisci, ligaments, tendons, synovium, and capsule) that function as an organ system during rapid growth, adult life, and aging. As with other organ systems, the integrity and function of the knee is controlled locally and systemically by different hormones, including sex hormones (estrogens, androgens, and progesterone) and their metabolites. Degenerative changes in the knee result in osteoarthritis (OA) in women and men, but there are sex-dependent differences in the incidence and severity of the disease that vary with hormonal status. The focus of preclinical OA studies has been on *in vitro* and *in vivo* male models, which neglects both the sexual dimorphic component of the disease and the potential effects of sex hormones on the incidence and progression of knee OA, as well as on the regenerative potential of affected cartilage. Recent studies examining the role of migratory chondrogenic progenitor cells in the repair of OA cartilage show that there are sex differences in the response of these cells to estrogen and testosterone [1]. Premenopausal levels of estrogen stimulate chondrogenesis in female progenitor cells, whereas testosterone stimulates chondrogenic differentiation of male progenitor cells. The purpose of this review is to summarize current knowledge on the role of sex hormones in modulating knee homeostasis and how changes in the sex hormone milieu during development and aging contribute to the incidence, severity, and progression of knee OA.

#### Synthesis of sex steroids

**Figure 1:**
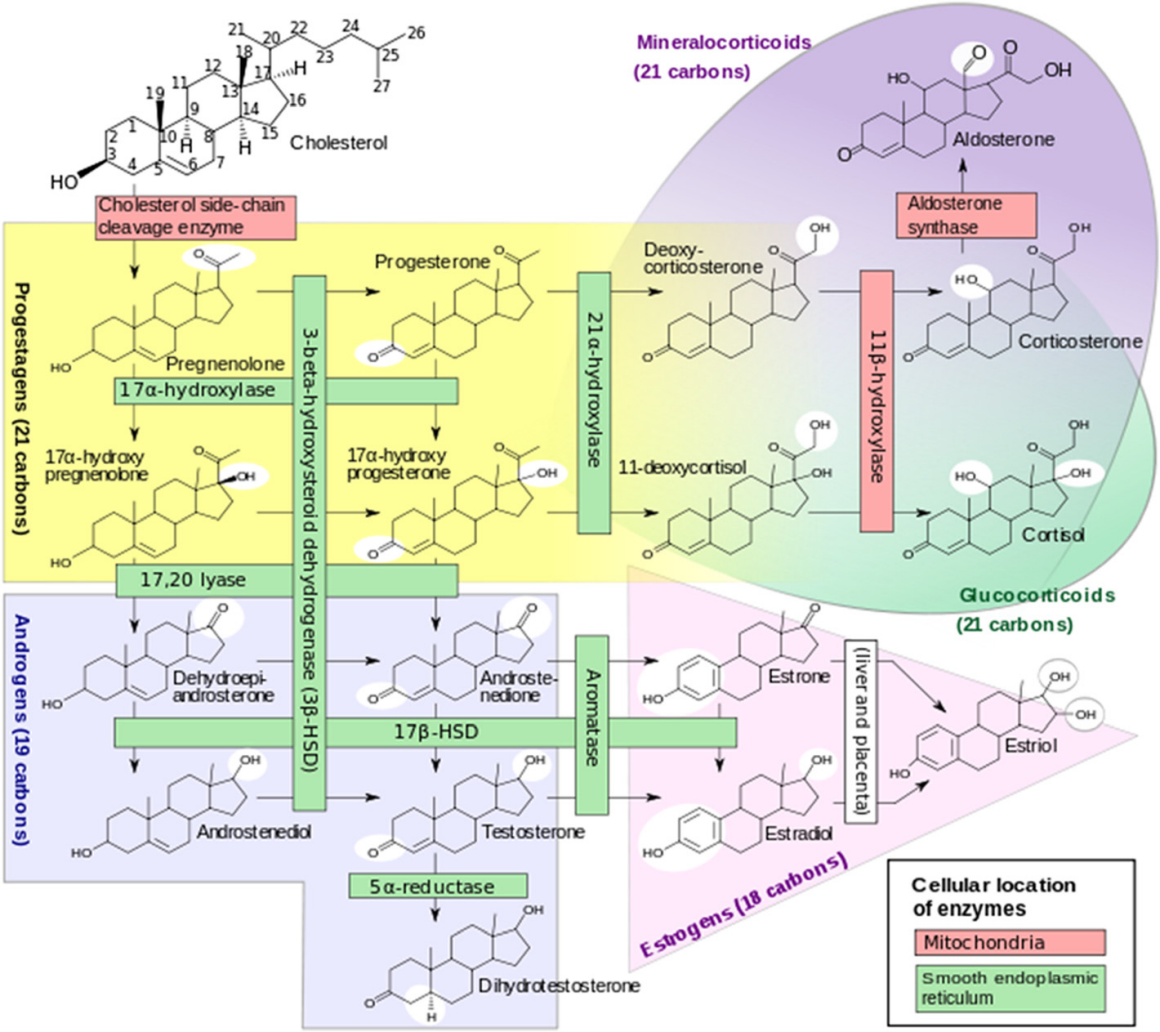
**Synthesis of sex steroids (**http://en.wikipedia.org/wiki/File:Steroidogenesis.svg#file**).** The schematic indicates the various enzymatic pathways for synthesis of different biologically active steroid metabolites of cholesterol as the starting molecule. The various pathways are regulated in different tissues by the expression of specific enzymes that catalyze the metabolic conversions in mitochondria and smooth endoplasmic reticulum. Some tissues (e.g., adrenal glands, gonadal tissues) are the primary source of some of the biologically active steroid metabolites, whereas others can be made locally in tissues such as articular cartilage when appropriately stimulated.

Sex hormones are a group of steroids derived from cholesterol that share a number of characteristics. Estrogens, androgens, and progesterones possess a cholesterol backbone (Figure 1) that make them lipid soluble. The synthesis of sex steroids is both sex- and tissue-dependent. Sex steroids are produced primarily in the sex glands and function systemically as part of the endocrine system. The circulating levels of these hormones reflect sex differences in both gonadal secretion and peripheral metabolism, both of which are sensitive to the availability of precursors and cyclic changes in regulatory factors. Circulating levels also change with physiological status. Circulating estrogen is high around the time of birth in both males and females and lower in males than females during early growth and development. At puberty, testosterone levels increase in males, whereas estrogen and progesterone increase in females. At menopause, estrogen levels decrease in women to levels that are lower than circulating levels in men.

Sex steroids are produced in other tissues besides the gonads, including the connective tissues of the knee, where they serve autocrine/paracrine functions. There is also sexual dimorphism in the extra-glandular metabolism of sex steroids. In growth plate chondrocytes from males, for example, testosterone is predominantly metabolized by 5α-reductase, resulting in the production of DHT [2]. In growth plate cartilage cells from females, testosterone is predominantly metabolized by aromatase, resulting in the production of estrogens. Thus, sex-specificity may be conferred not only by gonadal hormone secretion, but also by steroidogenic enzyme activity in knee tissues.

#### Mechanism of action

Sex steroids bind to cytosolic receptors, which then dimerize and translocate to the nucleus to modulate gene transcription [3]. Steroid hormones also act at the level of the cell membrane by stimulating signal transduction pathways traditionally associated with G-protein coupled receptors [4,5]. Cells in knee articular cartilage, as well as bone and muscle, in both males and females express receptors for estrogens, androgens, progesterone, and their metabolites [1], suggesting that both sexes can respond to hormones traditionally associated primarily with one sex. The numbers of receptors, their affinity for a hormone or hormone metabolite, or the presence and function of components of a particular signaling pathway may vary in a sex-specific manner [6-8]. In addition, individual tissues in the knee may be regulated by sex hormones in a differential manner that may influence the function of the knee as an organ system.

#### Contribution of sex steroids to knee OA

The research summarized in the next section indicates that differences in the regulation of individual tissues of the knee by sex hormones may influence the incidence and severity of OA. There are a number of potential mechanisms that may mediate this effect (Table 1). The goal of this paper is to present a synopsis of the current state of knowledge of sex hormone regulation of knee tissues and to identify gaps in knowledge.

**Table 1:** Complexities in hormonal regulation of knee tissues contribute to sex-differences in severity and incidence of knee OA

●	Changes in systemic levels of sex steroids.
●	Changes in local production of hormones or their metabolites.
●	Presence of hormone receptors, their affinity for ligand, or components of their signaling pathway.
●	Distribution of hormone-responsive cells in individual tissues of the knee.
●	Physiological status, including age and the presence of disease or tissue injury.

#### Mechanisms of sex hormone function

Sex hormones act via traditional steroid hormone receptor-mediated mechanisms and via membrane-associated rapid signal transduction pathways. How these receptor-dependent mechanisms impact knee homeostasis is discussed briefly.

#### Estrogen Receptors (ER)

ERα and ERβ are the products of different genes. Both receptor forms are found in estrogen responsive cells, but often at differing levels [9]. ERα is present in two isoforms, ERα66 and a truncated form, ERα46. In addition, a third form of ERα has been identified, ERα36, which is encoded by a different gene. ER-estrogen complexes can interact with DNA directly via estrogen-response elements (EREs) after dimerization or via complexes with other nuclear proteins to interact with other DNA sites such as AP-1 [10]. The concept of ligand-independent and ligand-dependent actions of ERs is further substantiated by the presence of ER variants in bones, termed estrogen-related receptors (ERR), which lack the ligand-binding domain in their structure [11,12]. Thus, the presence of hormones is not required for ERs and their respective ligands to influence tissues such as those of the knee.

ERα and ERβ have also been found associated with the plasma membranes of estrogen responsive cells [9,13-15], where they play a role in rapid responses to the hormone and membrane-associated signaling pathways [16]. In human articular chondrocytes, membrane-mediated signaling increases cell proliferation and cartilage matrix production [17,18]. This effect of estrogen is observed primarily in cells from female donors, but the reasons for this sex-specificity are not well understood. In growth-plate chondrocytes, there are differences in the number of total receptors (females > males) [15,18] and there are differences in ERα36 that may impact its mechanism of action (ElBaradie, Schwartz, Boyan et al. unpublished data), but it is not known if similar differences exist in articular chondrocytes. The actual scope of influence of the plasma membrane-associated receptors in articular cartilage is only now emerging, but the field is progressing rapidly [19] and it may prove important in designing therapeutic approaches for treating knee OA.

#### Androgen receptors

The androgen receptor (AR) is also a member of the nuclear hormone receptor family. There are traditional nuclear ARs and plasma membrane-associated ARs [20,21], similar to the ERs. Furthermore, some AR variants lacking a ligand-binding domain have been detected in both normal and cancerous tissues [22-24]. As both males and females produce testosterone and its metabolites, it is likely that the AR also influences some functions in females [25]. Recent studies suggest, however, that the cellular responses may be sex-specific. Female growth plate chondrocytes convert testosterone to estrogen, whereas male growth plate chondrocytes convert it to dihydrotestosterone (DHT) [2], and only male cells exhibit rapid membrane-associated responses to DHT [26]. The sex of the cell, therefore, may determine the outcome of ligand receptor binding more than simply the presence of the receptors in the cells.

#### Progesterone receptors

Traditional progesterone receptors (PR) exist as two natural genetic variants (PR-A and PR-B). Both variants can be expressed in the same cell, but the ratio of PR-A/PR-B can vary between cells [27], similar to that for ERα/ERβ [28]. In addition, membrane-associated forms of PR have been detected on various cells [29]. The widespread occurrence of PRs and conjugated ligands indicates they may have a more general influence on cell metabolism and function in connective tissues, such as those of the knee. This conclusion has been implicated from the study of menstrual cycle-associated alterations in knee joint laxity in subsets of young females [30].

#### Sex hormone receptor deficiencies

Much information about the function of sex hormone receptors has been gleaned from either naturally occurring mutations in preclinical models, or from induced deficiencies via generation of knockout (KO) mice [31]. Preliminary studies using the double ERα/ERβ KO mouse have not revealed histological differences in knee tissues (Hart et al., unpublished). There do not appear to be alterations to collagen fibril diameters and density in the ligaments of these mice, suggesting that the deficiencies do not exert a detectable impact on collagen assembly, but the biomechanical properties of the ligaments have not been assessed. These mice do possess ERα36, however, raising the possibility that this ER-variant is important in defining sex differences in response to estrogen. How this impacts human knee OA is unknown.

#### Sex hormone receptors and the knee

As detected by either immunolocalization or assessment of mRNA levels, expression of sex hormone receptors has been reported in nearly all tissues of the knee. Studies comparing chondrocytes in the growth plates of mice and rats and on articular chondrocytes from rats and human donors indicate that ERs and ARs are present in cells from both sexes [1,18,32]. Moreover, they exhibit comparable binding affinities for their ligands. However, the numbers of ER receptors are reduced in males relative to females [18]; it is not known if a comparable difference in ARs also exists. Unfortunately, quantification studies have usually not been done at a functional (e.g., protein level) level for most tissue-based studies. Thus, mRNA detection does not guarantee translation and immunolocalization does not necessarily detect intact receptors. One additional limitation is the lack of availability of an appropriate spectrum of reagents for some species of receptors.

Despite these limitations in our understanding of the regulation of sex hormone receptors in many tissues of the knee, it appears that receptor levels are dynamic and not static. For example, mRNA levels for ERs in tissues of the rabbit knee, as detected by RT-PCR, can be significantly influenced (significant elevations or depressions) by pregnancy, ligament injury, ovariohysterectomy, development of osteoarthritis following ACL transection, and during development of antigen-induced arthritis [33]. Furthermore, ER and PR mRNA levels appear to be regulated independently, indicating that separate elements influence expression levels. Although human tissues have not been assessed as extensively as those of preclinical models, the associations likely extend across species.

Although it is difficult to examine hormone receptors in intact tissues of the knee, due mainly to the paucity of cells in these matrix-rich environments, such studies are possible by using cells derived from the tissues. These types of studies have focused mainly on cells from bone (osteoclasts and osteoblasts), articular cartilage (chondrocytes) [17], skeletal muscle, synovial cells (type A and type B), tendon (tenocytes, paratenon cells), ligaments (ligament proper and epiligament cells) [34], and to a lesser extent cells from menisci and joint capsule. There are virtually no direct comparisons between cells of multiple tissues and only limited studies between cells from females and males. It is likely that differences in receptor levels exist, as noted above.

#### Sources of ligands for sex hormone receptors

Ligands for sex hormones can be generated by specific organs (endocrine system) and transported by the circulation to reach various target tissues, produced locally by cells such as macrophages and synthesized locally by metabolic alteration through expression of specific enzymes that are unique to various tissues and cells [35]. As many of the natural ligands are modified from common precursors via interactive pathways, the availability of ligands in males and females depends on the expression of specific enzymes. Molecules that can interact with sex hormone receptors can also be acquired via the environment and diet (e.g., phytoestrogens) [36]. Selective estrogen-receptor modifiers (SERMs) are molecules that can differentially influence ERα vs ERβ [37]. For example, tamoxifen, a drug widely used to treat breast cancer [38], can have a positive effect on ERα activity in breast tissue, but it increases cancer risk via association with ERβ in the ovaries [39]. How such SERMs influence cells in knee tissues is largely unknown.

Ligand availability is critical; premenopausal women have higher levels of circulating estrogens than men, and men have higher circulating levels of testosterone than women [40]. Moreover, there are sex differences in local concentrations of these hormones among those tissues that are able to synthesize sex steroids locally. Local production of ligands that can interact with sex hormone receptors with varying affinity and outcomes can be achieved in tissues such as bone and cartilage. Growth plate chondrocytes produce estrogen and DHT locally and at levels as high as 10^-8^ M [2,41], four orders of magnitude higher than in the blood. Similarly, articular chondrocytes also exhibit the ability to metabolize testosterone to estrogen and DHT [42,43]. The observation that cartilage cells respond to these concentrations via membrane-associated signaling pathways suggests that sex differences in these cells are likely due to local production and signaling, although it should be noted that sex differences have also been observed using concentrations of sex hormones at levels comparable to those found in blood [1]. How such differences could contribute to risk for loss of knee function remains to be determined.

Most studies on the influence of sex hormones on connective tissues of the knee have tended to assume the effects are direct. For example, investigators have either added specific agonist or antagonist ligands to *in vitro* cell populations and observed the outcomes on cell metabolism or explant behavior, or replaced the endocrine products *in vivo* that are lost due to surgical castration (removal of ovaries/uterus, testicles). In addition, many of these studies use concentrations of the specific ligands that exceed circulating physiologic levels.

A second issue regarding the influence of sex hormone effects on the connective tissues of the knee is that all tissues of the knee, with the exception of articular cartilage, are innervated and vascularized. Therefore, the influence of sex hormones from the endocrine system, which are transported by the microvasculature to the target cells within the tissues, could influence the knee tissues via the endothelial cells of the vasculature. Alternatively, the modulation could be achieved either directly or indirectly by neuropeptides via the neuroendocrine system. The exception to these possible indirect mechanisms is articular cartilage (avascular and aneural), where hormones produced by the endocrine system must diffuse into the tissue or be produced locally.

#### The balance between sex hormone receptors and their ligands

Although both males and females express estrogen, progesterone, and androgen receptors and make various ligands for those receptors, they do so at different levels and via distinct regulatory pathways. The levels for specific ligands change across the lifespan and are matched by changes in receptors, as well as intracellular proteins that can function as co-activators/co-repressors [44]. These adjustments result in function being related to the balance between the involved elements, and menopause corresponds to a change in the balance between estrogen, progesterone, and testosterone-related ligands produced systemically and locally rather than just a loss of specific endocrine components. However, the impact of systemic loss of endocrine contributions (e.g., menopause) to this balance likely varies across tissues due to differences in the expression levels of hormone receptors and the potential to produce ligands locally.

Studies in preclinical rabbit models have indicated that levels of estrogen receptors in connective tissues of the knee can be influenced by injuries to the knee (ACL transection, MCL transection) and hence, their levels are dynamic and not static in the cells of these tissues. Thus, ER levels in tissues of the knee are differentially influenced by systemic and locally produced ligands. Therefore, in females where hormone levels can fluctuate over the menstrual cycle, during pregnancy, and following menopause, cells in connective tissues of the knee would likely experience a more varying hormonal environment than age-matched males.

#### Sex differences in hormonal regulation of knee tissues and function

The discussion in the preceding sections focused on the biochemistry and cell biology of sex steroids and their receptors. This section examines how such hormone/hormone receptor systems impact specific tissues of the knee, and how such an influence may contribute to sex differences in the development of OA.

#### Anterior cruciate ligament

The incidence of knee injuries, particularly those involving the anterior cruciate ligament (ACL), is more common in female athletes than males for a number of sports [45-49]. The prevalence of ACL injuries experienced by females in such sports as soccer, basketball, and handball is 5- to 10-times greater than in males, and most are non-contact injuries. The peak age of increased risk in females is 16–20 years, which is a time of growth and development underlying puberty. It remains unclear how changes in systemic sex hormones may influence susceptibility to ACL injury.

Cyclic sex hormone-dependent joint laxity has been implicated in the etiology of ACL injury in young women, although this is not a uniform finding [30,49,50]. The mixed findings may reflect differences in how the studies were conducted or differences in the populations examined. The latter hypothesis is supported by the observation that menstrual phase-associated changes in knee function were apparent in some females and not others, even though changes in estrogen and progesterone were comparable [31,51,52]. When changes in knee function were observed, they usually involved knee joint laxity and muscle function. Because joint laxity is likely more critical when the extra-articular ligaments and the capsule experience low loads, modulation of joint laxity during the menstrual cycle may influence positioning and function of the knee during high-risk maneuvers [53,54].

There is an urgent need to identify the mechanisms and individuals at risk for ACL injury because approximately 50% of those who experience such an injury develop overt knee OA irrespective of whether or not the ACL is surgically reconstructed using conventional orthopedic procedures [55]. The reason for the prevalent progression to knee OA after ACL injury is not known. It is clear that ACL rupture can lead to altered knee stability, but it is not known if the injury is exacerbated by previous menstrual cycle-associated alterations in joint laxity, or if the downstream effects of the injury itself dominate the eventual outcome. Potential contributing factors include collateral tissue damage (e.g., meniscal injury) at the time of the ACL injury, injury-associated changes in muscle function, and continued menstrual cycle-associated exacerbations of injury-induced changes to knee function.

Ligament injury appears to play a role in the sex-dependent development of knee OA, but it is less clear whether there are sex differences in OA development early after a knee injury (10–15 years) [56-58]. Sex differences in outcomes after ACL injury and reconstruction have been reported [59], suggesting that there may be sex differences in outcomes following knee injury as well. However, there is no consensus on this possibility [60].

#### Joint hypermobility syndrome and knee OA

The prevalence of joint hypermobility syndrome (JHS), formerly called benign JHS, is associated with a generalized condition where individuals have lax joints (e.g., “double jointed”) and is ~5 times more common in females than in males. JHS has been associated with mutations in some matrix molecules [61-63], but non-syndromic forms have also been identified. There are also population differences in the incidence of JHS [49,61], further complicating the identification of sex differences in its prevalence. Females with the syndrome exhibit a low-grade synovitis, possibly related to accumulated micro-injury of the knee due to load-related damage [64]. Because the control of inflammatory processes in females can differ from those in males, the significance of synovitis in the knees of females with joint hypermobility syndrome remains to be clarified.

Genetics presumably contribute to the development of JHS, but non-genomic or epigenetic variables must also be involved because concordance was less than 100% in identical twins [65]. It is not known whether females with JHS experience modulation of joint laxity during the menstrual cycle or pregnancy. Interestingly, an inverse association between joint hypermobility and knee osteoarthritis has been reported [66]. Although this finding may appear counterintuitive, the study involved multiple cohorts and suggests that it is necessary to reassess concepts related to inflammation and joint positioning with knee OA development.

#### Direct effects of sex hormones on articular cartilage

**Figure 2:**
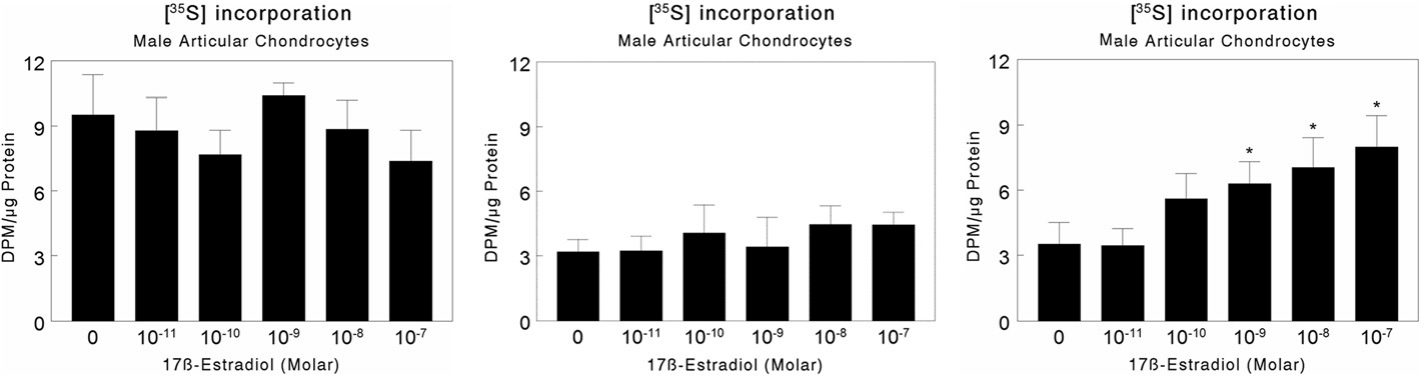
**Variable response of male human articular chondrocytes to 17β-estradiol.** Chondrocytes were isolated from three male donors (<30 years) with no evidence of OA. Confluent cultures of second-passage cultures were treated with 10^-8^ to 10^-10^ M 17β-estradiol for 24 hours and proteoglycan production was measured as a function of ^35^S-sulfate incorporation into sulfated glycosaminoglycans, a hallmark of cartilage extracellular matrix. Data are means ± SEM, N = 6 independent cultures per variable. *p < 0.05, treatment v. control. Cells from two of the donors exhibited no response to the hormone in two different sets of experiments, whereas cells from one of the donors were responsive to the hormone. Data are provided courtesy of Ramsey Kinney, MD, PhD, Department of Orthopaedics, Emory University School of Medicine, Atlanta, GA.

Sex steroids appear to have complex effects on articular cartilage via direct actions on chondrocytes and contribute to OA-associated changes, but many details remain to be determined [67]. Human articular chondrocytes express estrogen receptors ERα and ERβ [68,69], indicating that they are estrogen-responsive cells. In organ cultures from mature rat articular cartilage, estrogens reduce chondrocyte proliferation and increase chondrocyte hypertrophy based on thinning of the cartilage and production of type X collagen [70]. In contrast, 17β-estradiol results in proliferation of chondrocytes from adult humans and stimulates production of type II collagen and aggrecan [68]. Furthermore, premenopausal concentrations of 17β-estradiol prevent telomere shortening in articular chondrocytes whereas post-menopausal concentrations do not [71], supporting the hypothesis that estrogens protect against OA via direct actions on chondrocytes. Moreover, 17β-estradiol reduces expression of matrix metalloproteinases associated with cartilage matrix degradation in OA [72]. Although cells from male and female donors have ERα and ERβ, female cells are more responsive to 17β-estradiol than male cells [17,69]. Male cells can respond to high concentrations of the hormone, but this is not a consistent finding. When human articular chondrocytes were examined for rapid activation of protein kinase C in response to 17β-estradiol, no increase in enzyme activity was observed in male cells, whereas all female chondrocytes responded. In contrast, synthesis of extracellular matrix proteoglycan, which depends on traditional nuclear receptor binding, was variable with 17β-estradiol having a stimulatory effect on cells from one of three male donors (Figure 2). This suggests that the sex-specific responses to the hormone are mediated via membrane-associated forms of the estrogen receptor.

Less is known about the direct effects of testosterone on articular chondrocytes, but cells from both male and female donors express receptors for testosterone and DHT [72], indicating that these cells are sensitive to regulation by androgens. In addition, articular chondrocytes possess the ability to synthesize estrogens and androgens [73], further supporting the hypothesis that sex steroids act directly on these cells. Several studies found that androgens protect against cartilage degradation in rheumatoid arthritis, suggesting they play a similar role in OA, but it is not known if this is a direct effect of testosterone or DHT or if it is due to estrogen produced locally via aromatase action.

The discrepancies among different studies are likely due to a variety of issues: whether male or female cells and tissues were used, the age of the donors, the species of donor, the concentration and length of exposure to the hormone, the maturation state of the cells in culture, whether cells came from healthy or osteoarthritic tissue, and the site within the articular cartilage from which the cells were isolated. The lack of consistency among studies has made it difficult to assess the role of sex steroids in the etiology of OA.

#### Menopause-associated risk for development of knee OA

Prior to menopause, the incidence of idiopathic knee OA is similar in women and men. After menopause, however, the prevalence is much greater in females than age-matched males [74,75]. These observations indicate that withdrawal of ovarian hormones increases risk for the development and progression of overt knee OA in women. However, not all postmenopausal women develop knee OA and it is not clear what distinguishes those who do from those who do not. To address this gap in knowledge, some studies have begun to investigate systemic aspects of molecular changes in sex hormone metabolism after menopause [76], as well as direct and indirect effects of estrogens on cartilage in various populations [77].

A widely used approach to identify the influence of menopause on connective tissue homeostasis has been to examine the influence of surgical menopause (surgical removal of the ovaries or ovaries + uterus) on connective tissues of the knee and bones. Results have indicated that the consequences at the mRNA level in the rabbit are not the same for all connective tissues of the knee [78], there is a rapid onset of osteopenia in mice and rats [79,80], and surgical menopause in primates leads to osteopenia [48]. However, the decline in systemic estrogens in primates did not result in changes in the response of the patellar tendon or ACL to mechanical load even after two years, which suggests that postmenopausal reductions in serum estrogens in primates also may not influence homeostasis of connective tissues of the knee equally.

One feature of this work, especially the studies of osteoporosis in human populations, is that the loss of bone density and structure is variable across individuals. Although a genetic component is likely involved, loss of ovarian function after menopause leads to different rates of bone loss. Because variable loss of bone mass has also been observed in astronauts during exposure to microgravity [81], the rate of bone loss is likely influenced by mechanical factors and the balance between anabolism and catabolism in a tissue [82]. If the findings in bone translate to other tissues of the knee, this may help explain the individual variations in the development and progression of knee OA in females based on the contribution of the sex hormone-hormone receptor set point (e.g., levels and type of hormone receptor, hormone-receptor modulating proteins, local metabolism of hormones) in each tissue. Such variation may be relevant to knee OA because bone changes, particularly subchondral bone, during the development of OA [83,84].

A limitation of the surgical menopause approach is that it is usually performed on young adult animals and as such does not account for the superimposed age-related changes on the knee tissues. For instance, it has been reported that loss of knee cartilage is much greater in 50- to 79-year-old women than in age-matched men [85], which suggests that there are distinct effects of age and menopause. Therefore, the surgical menopause models using younger animals do not capture the age-associated adaptations in the tissues, which limits the interpretation of the findings.

#### Sex differences in inflammation

OA is an inflammatory disease [86,87] and women tend to have more robust inflammatory and immune responses than males [88]. The greater prevalence of knee OA in older women than men may be due, in part, to development and persistence of inflammatory cytokines in the knee. Such effects may be secondary to the influence of hormones. For example, postmenopausal reductions in circulating estrogen are associated with an increase in the production of inflammatory cytokines, such as interleukin-6 (IL-6) [89]. The possibility that hormone-associated changes in systemic and tissue-specific inflammatory cytokines can influence the risk of knee OA should be addressed in future studies so that it is possible to identify those individuals at higher risk.

## Conclusion

A major research gap in this area is the lack of relevant and reproducible cell culture and animal models that can be used to describe the etiology and progression of knee OA disease and to understand the underlying mechanisms and the role of sex hormones. There is little understanding of how the inflammatory process contributes to knee OA development and progression, and whether such effects are sex-specific. It is clear that risk level varies among women, but that women are at higher risk than males. Sex hormones and hormonal variation have been implicated in knee OA risk, but much of the data are correlative and the underlying cellular or molecular mechanisms are unknown. In parallel with the recognition of sex-based differential risks, however, a literature has evolved that provides new clues on how sex hormones and their receptors can influence connective tissues. What is missing is the integration of information on hormone effects on connective tissues and cells with the augmented risk in females for loss of knee function via OA, which will be needed to identify sex-specific mechanisms and therapeutic targets for prevention and treatment.

### GAPS In our knowledge

A number of investigations by many groups have revealed details on how sex hormones (local and systemically produced) and their receptors can influence cells of connective tissues of the knee complex. However, the incorporation of this new information into an understanding of how the findings contribute to risk for knee injury and degenerative disease, and the functioning of the knee in a variety of biomechanical environments remains elusive. Thus, there is a real gap in translating the basic biomedical findings to the clinical setting. Of critical importance is the use of such information in the prevention of knee injury and further risk for degenerative loss of function. Only through such translation can risk be mitigated, and development of appropriate interventions fostered.

It is clear from the literature discussed that considerable knowledge has been generated over the past decade regarding the *potential* of connective tissues of the knee to generate and respond to sex hormones that *may* contribute to the observed sex differences in risk for loss of knee function and integrity due to injury and degenerative diseases such as OA. However, there remain significant gaps in our understanding of how sex steroid contribute to knee OA (Table 2). Moreover, the use of this information to establish cause-and-effect relations between the two sets of observations remains the most significant gap in our understanding of the cellular and molecular basis for the risk.

**Table 2:** Research gaps in our understanding of sex differences in OA

1.	Lack of good epidemiologic information on heterogeneity in both the female and male populations with respect to hormonal status v. incidence and severity of OA.
2.	Need for basic science studies on the etiology of OA in males v. females.
3.	Lack of valid preclinical in vitro and in vivo models of osteoarthritis.
4.	Absence of studies specifically examining sex hormone mechanisms in cells from knee tissues.
5.	Uncertainty about the role of inflammation at the cell, tissue, and organ levels.
6.	Need to determine if the risk for loss of knee function and integrity in females is restricted to only the knee or if sex-specific changes in other tissues play a role.

## Abbreviations

ACL: Anterior cruciate ligament; AR: Androgen receptor; DHT: Dihydrotestosterone; DNA: Deoxyribonucleic acid; ER: Estrogen receptor; ERS: Estrogen-response element; ERR: Estrogen-related receptors; IL-6: Interleukin-6; JHS: Joint hypermobility syndrome; KO: Knockout; MCL: Medial collateral ligament; mRNA: Messenger ribonucleic acid; OA: Osteoarthritis; PR: Progesterone receptor; RT-PCR: Real-time polymerase chain reaction; SERMs: Selective estrogen-receptor modifiers.

## Competing interests

The authors declare that they have no competing interests.

## Authors’ contributions

All authors contributed to the development of the review. All authors read and approved the final manuscript.
